# Exploring the Causal Links Between *Toxoplasma gondii* Infection and Risk of Brain Tumors: A Bidirectional Mendelian Randomization Analysis

**DOI:** 10.1002/brb3.71239

**Published:** 2026-01-29

**Authors:** Pengqiang Shi, Gangao Wei, Zhenwei Li, Baoshun Du, Guodong Zhang, Jiaqi Zhang, Yungang Wang, Yunchao Chen, Zhang Cheng, Zhenguo Cheng

**Affiliations:** ^1^ Department of Neurosurgery Xinxiang Central Hospital Xinxiang China; ^2^ The Fourth Clinical College of Xinxiang Medical University Xinxiang China; ^3^ Xinxiang Key Laboratory of Cerebrovascular Disease Metabolomics Xinxiang China

**Keywords:** brain cancer, GSMR, MR, *T. gondii*

## Abstract

**Background:**

*Toxoplasma gondii* (*T. gondii*) is a ubiquitous protozoan parasite capable of establishing lifelong latent infections in the central nervous system. Previous epidemiological studies have suggested a potential association between *T. gondii* infection and an increased risk of brain cancer, but the causal relationship remains unclear.

**Methods:**

We conducted a bidirectional Mendelian randomization (MR) study to assess the causal relationship between *T. gondii* infection and brain tumor risk. Genetic instruments for *T. gondii* seropositivity were derived from a genome‐wide association study (GWAS) in the UK Biobank, while genetic data for brain tumors were obtained from the FinnGen R12 dataset. Standard MR methods, including inverse‐variance weighted (IVW), weighted median, and MR‐Egger, were applied to infer causality, with generalized summary Mendelian randomization (GSMR) used for further validation. Sensitivity analyses, including heterogeneity and pleiotropy assessments, were performed to ensure robustness. Additionally, reverse MR analyses were conducted to evaluate whether brain tumors influence genetic liability to *T. gondii* seropositivity.

**Results:**

Our MR analyses found no evidence of a causal relationship between genetic liability to *T. gondii* seropositivity, as indicated by P22 and SAG1 antibody levels, and the risk of brain tumors. Across all tumor subtypes, IVW, weighted median, MR‐Egger, and GSMR analyses consistently yielded non‐significant results. However, reverse MR analysis suggested that genetic liability to malignant brain tumors is associated with increased odds of *T. gondii* seropositivity. For P22, a strong association was observed across methods (IVW: OR = 1.234, *p* = 0.004; GSMR: OR = 1.228, *p* = 0.006). In contrast, for SAG1 the evidence was weaker, with IVW indicating a suggestive association (OR = 1.094, *p* = 0.048) and GSMR showing a borderline association (OR = 1.088, *p* = 0.052). Sensitivity analyses confirmed the robustness of these findings, with no evidence of heterogeneity or pleiotropy. No significant associations were observed for meningioma, glioblastoma, or benign brain tumors.

**Conclusion:**

Our study provides no evidence for a causal relationship between genetic liability to *T. gondii* seropositivity and brain tumor risk. However, reverse MR suggests that genetic liability to malignant brain tumors may be associated with increased odds of *T. gondii* infection.

## Graphical abstract

Mendelian randomization (MR) analysis demonstrated that genetic liability to malignant brain tumors was significantly associated with increased odds of *Toxoplasma gondii* (*T. gondii*) seropositivity, suggesting that individuals genetically predisposed to brain tumors may have a higher susceptibility to *T. gondii* infection.

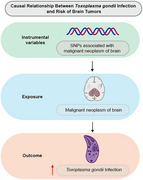

## 1 Introduction

*Toxoplasma gondii* (*T. gondii*), an obligate intracellular parasite, is one of the most prevalent protozoan infections in humans, with an estimated global seroprevalence exceeding 50% in certain populations (Torgerson and Mastroiacovo 2013; Gelaw et al. 2024; Bisetegn et al. 2023). Transmission occurs primarily through ingestion of tissue cysts from undercooked meat, ingestion of oocysts from contaminated water or soil, or congenital transmission (Almeria and Dubey 2021). While *T. gondii* infection is typically asymptomatic in immunocompetent individuals, accumulating evidence suggests a potential link between chronic infection and neurological disorders, including schizophrenia, epilepsy, and neurodegenerative diseases (Daher et al. 2021; Virus et al. 2021). Beyond these neurological effects, there is growing interest in whether chronic *T. gondii* infection may contribute to the risk of brain tumors, although this relationship remains poorly understood.

Brain tumors constitute a heterogeneous group of malignancies with varying etiologies and prognoses (DeAngelis 2001; Weller et al. 2015). Several epidemiological studies have proposed an association between *T. gondii* seropositivity and increased glioma risk (Abdollahi et al. 2022; Hodge et al. 2021; Thomas et al. 2012), potentially mediated by chronic neuroinflammation, immune modulation, and direct oncogenic effects of the parasite. Nevertheless, observational studies are inherently susceptible to confounding factors and reverse causation, limiting the ability to establish a causal relationship. In addition, clinical diagnostic challenges further complicate interpretation, as infectious mimickers such as brain abscesses can radiologically and symptomatically resemble brain tumors, potentially leading to misclassification in epidemiological studies (Choucha et al. 2025). These limitations highlight the need for robust causal inference approaches to clarify the relationship between *T. gondii* infection and brain tumor risk.

Mendelian Randomization (MR) offers a robust framework to infer causality by leveraging genetic variants as instrumental variables (IVs) to assess the association between an exposure (e.g., *T. gondii* infection) and an outcome (e.g., brain tumor) (Sanderson et al. 2022). This method minimizes confounding and reverse causation by exploiting the random allocation of alleles during meiosis (Emdin et al. 2017). Additionally, bidirectional MR allows for the assessment of potential reverse causality, distinguishing whether *T. gondii* infection is a risk factor for brain tumor or whether brain tumor predisposes individuals to infection, for instance, through immunosuppression‐related susceptibility. However, conventional MR approaches often assume independence among genetic instruments and may be influenced by pleiotropy, potentially biasing causal estimates. To address these limitations, generalized summary data‐based MR (GSMR) has been developed as a refined extension that improves the efficiency and robustness of causal inference. By accounting for linkage disequilibrium (LD) among genetic variants, GSMR enhances the power of analysis while reducing bias. Additionally, it incorporates the Heterogeneity in Dependent Instruments (HEIDI) test, which detects and removes pleiotropic single‐nucleotide polymorphisms (SNPs) that could violate MR assumptions (Zhu et al. 2018; Lin et al. 2019).

In this study, we applied bidirectional MR and GSMR to systematically evaluate the potential causal relationship between *T. gondii* infection and brain tumor risk. By integrating GWAS summary statistics, we aimed to assess the direction of causality and estimate the effect size of this association. This approach allows us to investigate whether genetic liability to *T. gondii* seropositivity influences brain tumor susceptibility or, conversely, whether genetic predisposition to brain tumors affects *T. gondii* antibody levels, thereby providing insights into potential interactions between parasitic exposure and neuro‐oncology.

## 2 Method

### 2.1 Study Design

**FIGURE 1:**
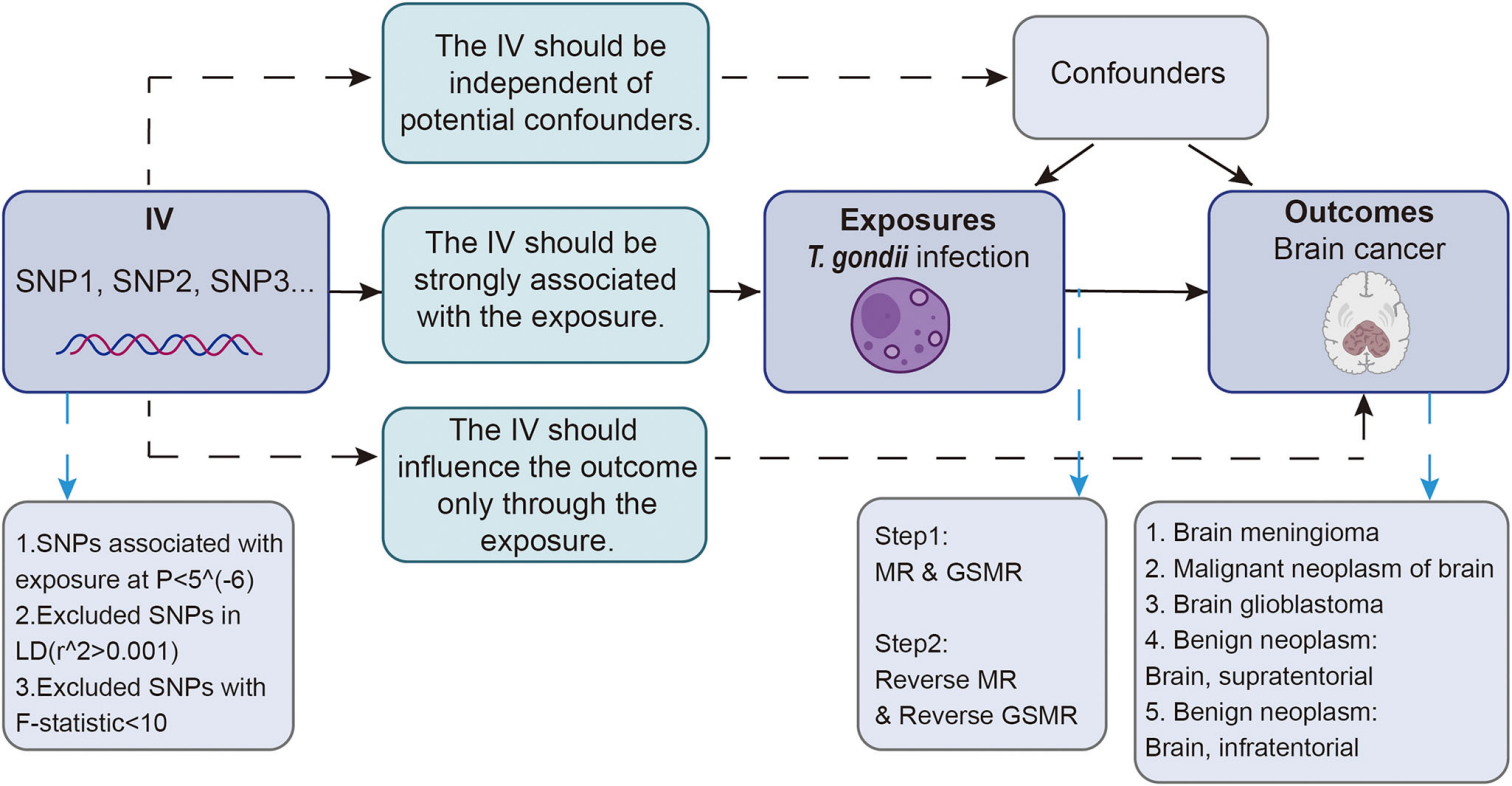
**Study design for investigating the causal relationship between *T. gondii* antibody seropositivity and the risk of brain tumor**. This figure outlines the two‐step approach employed to examine the causal relationship between *T. gondii* infection and brain tumor risk using conventional MR and GSMR. In Step 1, genetic proxies for seropositivity to two anti‐*T. gondii* antibodies (P22 and SAG1) were used as IVs to assess the causal effect of *T. gondii* infection on the risk of multiple brain tumor subtypes, including meningioma, glioblastoma, malignant neoplasm of the brain, and benign tumors, further stratified by supratentorial and infratentorial locations. In Step 2, bidirectional MR and GSMR analyses were performed to explore reverse causality, investigating whether brain tumors, especially malignant ones, may predispose individuals to T. gondii seropositivity through tumor‐mediated immune suppression or treatment‐induced immunomodulation. The analysis adhered to the MR‐STROBE checklist for robust quality control, pleiotropy assessment, and sensitivity analyses to ensure the reliability of the findings. **Abbreviations**: GSMR, generalized summary Mendelian randomization; IV, instrumental variable; MR, Mendelian randomization; SNP, single nucleotide polymorphism.

This study was designed in two complementary steps to investigate the causal relationship between genetically predicted *T. gondii* seropositivity and the risk of brain tumors using conventional MR and GSMR (**Figure** **1**). To ensure the validity of the MR approach, we adhered to three core instrumental variable assumptions (Emdin et al. 2017). First, the relevance assumption stipulates that selected IVs are strongly associated with genetically proxied seropositivity to anti*‐T. gondii* antibodies. Second, the independence assumption requires that IVs are independent of potential confounders influencing the association between *T. gondii* antibody seropositivity and brain tumor risk. Third, the exclusion restriction assumption stipulates that IVs affect brain tumor risk exclusively through genetically predicted *T. gondii* seropositivity, not via alternative pathways.

In the first step, we leveraged genetic proxies for seropositivity to two anti‐*T. gondii* antibodies (P22 and SAG1) as IVs to assess whether genetically predicted *T. gondii* seropositivity causally influences the risk of brain tumors. We examined multiple brain tumor subtypes, including brain meningioma, malignant neoplasm of the brain, glioblastoma, and benign neoplasms, further stratified by supratentorial and infratentorial locations. Given the distinct etiological and biological characteristics of these tumor types, we conducted subtype‐specific analyses to account for potential heterogeneity in the causal effects of genetically predicted *T. gondii* seropositivity. In the second step, we conducted bidirectional MR and GSMR analyses to explore potential reverse causality, assessing whether genetic liability to brain tumors, particularly malignant forms that may compromise immune function, is associated with genetically predicted *T. gondii* seropositivity. This is important because brain tumors may influence *T. gondii* serological response via tumor‐mediated immunosuppression or treatment‐induced immunomodulation. To ensure methodological rigor, we followed the MR‐STROBE checklist, implementing stringent quality control measures for IV selection, pleiotropy assessment, and sensitivity analyses to evaluate the robustness of our findings (Skrivankova et al. 2021). By integrating these analytical refinements, our study provides a systematic evaluation of the potential causal interplay between genetically proxied *T. gondii* seropositivity and the risk of brain tumors, offering insights into possible biological mechanisms linking parasitic exposure and tumor susceptibility.

### 2.2 Data Source

Genetic instruments for *T. gondii* seropositivity were derived from a GWAS investigating antibody‐mediated immune responses to infectious disease agents in the UK Biobank (UKB) cohort. The UKB is a large prospective study that recruited over 500,000 individuals aged 40–69 years from across the United Kingdom between 2006 and 2010 (Bycroft et al. 2018). Within this cohort, a subset of 8,976 participants of European ancestry underwent serological testing to assess their immune responses to various pathogens, including *T. gondii* (Butler‐Laporte et al. 2020). Seropositivity for *T. gondii* was assessed based on immunoreactivity to two key antigens, P22 and SAG1, using a multiplex serology approach. The study identified 1,308 individuals (14.6%) seropositive for the P22 antigen and 3919 individuals (43.6%) seropositive for the SAG1 antigen, with an overall *T. gondii* seropositivity rate of 27.3% (2449 individuals), defined as positivity for either antigen. The GWAS on seropositivity was conducted using median fluorescence intensity thresholds to classify individuals as seropositive or seronegative. The availability of high‐quality summary statistics from this study allows for robust MR analysis, facilitating the identification of genetic proxies for *T. gondii* seropositivity and enabling assessment of its potential causal relationship with brain tumor risk. P22 and SAG1 seropositivity were interpreted as markers of antibody‐mediated immune response to *T. gondii* exposure, which may reflect current or past infection but does not allow precise discrimination of the acute infection stage (Yang et al. 2024; Jeske et al. 2024).

Genetic association data for brain tumors were obtained from the FinnGen R12 dataset, a large‐scale, population‐based biobank study that integrates genomic and national health registry data from Finnish individuals (Kurki et al. 2023). Cases were identified using ICD‐coded diagnoses, while controls were selected from the general population, explicitly excluding individuals with any cancer diagnosis to minimize potential confounding due to shared genetic predisposition across malignancies. We analyzed multiple brain tumor subtypes, including brain meningioma (1835 cases, 377,674 controls), malignant neoplasm of the brain (1894 cases, 378,749 controls), glioblastoma (406 cases, 378,749 controls), benign neoplasm of the brain, supratentorial (569 cases, 499,779 controls), and benign neoplasm of the brain, infratentorial (201 cases, 500,140 controls). The FinnGen dataset provides a well‐powered and ancestrally homogeneous cohort, ensuring precise genetic effect estimation while minimizing population stratification. The large sample sizes, comprehensive registry‐based case ascertainment, and robust control selection strengthen the validity of our MR analysis, allowing for a rigorous evaluation of the causal relationship between *T. gondii* seropositivity and brain tumor risk. Data sources for MR analysis are summarized in **Table** **1**.

**TABLE 1:** GWAS data sources used for the MR study.

Exposure/outcome	Data source	Phenotype	Population	Sample size
Antibodies against *T. gondii*	Butler‐Laporte et al.	P22	European	1308
	Butler‐Laporte et al.	SAG1	European	3919
Brain tumor	FinnGen consortium	Brain meningioma, excluding all cancers	European	37,9509
	FinnGen consortium	Malignant neoplasm of brain, excluding all cancers	European	38,0643
	FinnGen consortium	Brain glioblastoma, excluding all cancers	European	37,9155
	FinnGen consortium	Benign neoplasm: Brain, supratentorial	European	50,0348
	FinnGen consortium	Benign neoplasm: Brain, infratentorial	European	50,0348

**Abbreviations**: MR, mendelian randomization; *T. gondii, Toxoplasma gondii*.

### 2.3 Selection of IVs

To ensure the validity of our MR analysis, IVs were selected based on the following stringent criteria. First, we identified SNPs strongly associated with seropositivity to *T. gondii* at genome‐wide significance (*p* < 5.0 × 10^−^^6^), a threshold selected to balance instrument strength and statistical power for serology‐based GWAS with limited sample size and in line with previous MR studies using the same data source (Wang et al. 2024; Zhong et al. 2023; Zhu et al. 2025). Second, to minimize LD effects, independent SNPs were selected using clumping (*r* ^2^ < 0.001, 10,000 Kb window). Third, to avoid weak instrument bias, we retained only SNPs with an F‐statistic > 10, ensuring sufficient instrument strength. For each selected SNP, the proportion of variance in *T. gondii* seropositivity explained (*R* ^2^) and the *F*‐statistic were calculated. The *R* ^2^ was determined using the formula: *R* ^2^ = [2 × (*β*_exposure)^2^ × EAF × (1 − EAF)] /[2 × (*β*_exposure)^2^ × EAF × (1 − EAF) + 2 × N × EAF × (1 − EAF) × (*SE*_exposure)^2^], where *β*_exposure represents the effect size of the SNP on *T. gondii* seropositivity, EAF is the effect allele frequency, N is the sample size of the GWAS, and SE_exposure is the standard error of the SNP‐exposure association (Shim et al. 2015; Papadimitriou et al. 2020). The F‐statistic was calculated using the formula *F* = *R* ^2^ × (N − 2)/(1 − *R* ^2^), where *R* ^2^ represents the proportion of variance explained by the SNP, and N denotes the GWAS sample size (Palmer et al. 2012). For the reverse causality analysis, we selected SNPs for brain tumor susceptibility from the FinnGen R12 GWAS, following the same criteria of genome‐wide significance, LD pruning, and *F*‐statistic filtering.

### 2.4 MR Analysis and Sensitivity Analyses

MR analysis was conducted to investigate the causal relationship between *T. gondii* seropositivity and the risk of brain cancer. In the primary MR analysis, the association of each genetic variant with brain cancer was weighted by its corresponding association with *T. gondii* seropositivity. These weighted associations were then combined using the IVW method, which provides an efficient and valid causal estimate under the assumption that the average pleiotropic effect across all IVs is negligible or zero (Burgess et al. 2013). To assess heterogeneity among SNP‐specific causal estimates, Cochran's *Q* test was performed. A *p*‐value < 0.05 was considered indicative of significant heterogeneity, suggesting potential violations of IVW assumptions, such as invalid instruments or substantial heterogeneity in genetic associations with brain cancer (Bowden et al. 2017). Importantly, the presence of heterogeneity does not invalidate MR results per se but indicates that causal estimates should be interpreted with caution and supported by complementary sensitivity analyses. Additionally, to account for potential pleiotropy, MR‐Egger regression was employed (Bowden et al. 2015). This method estimates an intercept term, which serves as an indicator of directional pleiotropy. A nonzero intercept (*p*‐value < 0.05) suggests the presence of unbalanced pleiotropy, warranting cautious interpretation of the causal estimates. In the context of our study, Cochran's *Q* test evaluates whether the causal estimates from individual SNPs are consistent, with significant heterogeneity implying that some instruments may violate MR assumptions (e.g., due to outlier SNPs or unmeasured confounders). A non‐significant result (*p* > 0.05) supports the validity of the IVW estimate by indicating low heterogeneity. Similarly, the MR‐Egger intercept tests for systematic bias from pleiotropic effects; an intercept close to zero (with *p* > 0.05) suggests no evidence of directional pleiotropy, reinforcing the reliability of the causal inference, whereas a significant intercept may indicate that pleiotropy is driving the association, potentially biasing the IVW results toward the null or away from it depending on the direction. These tests were applied to both forward and reverse MR analyses, with results reported in the main text and supplementary tables to ensure transparency and robustness assessment. Furthermore, reverse MR analysis was performed to explore potential causal effects of brain tumors on *T. gondii* seropositivity, assessing whether individuals with brain tumors are associated with higher *T. gondii* seropositivity.

### 2.5 GSMR Approach

To assess the potential causal relationship between *T. gondii* seropositivity and brain cancer risk, we implemented GSMR using the gsmr2 R package (repository: jianyanglab/gsmr2), with the gsmr2_beta parameter set to 1 for optimal causal effect estimation (Zhu et al. 2018). GSMR effectively accounts for LD and minimizes pleiotropic bias through systematic filtering procedures. For the selection of IVs associated with *T. gondii* seropositivity, we applied a clumping algorithm to identify independent SNPs reaching genome‐wide significance (*p* < 5 × 10^−^^6^) while ensuring low LD (*r* ^2^ < 0.05). Due to the prior clumping procedure (threshold *r* ^2^ < 0.05 within 1 Mb windows), the instrumental variables were considered independent, and therefore a diagonal LD matrix was applied in the GSMR model. To support variance estimation and maintain the robustness of the HEIDI‐outlier test, we specified a reference sample size of 7703 individuals from an ancestry‐matched European population, consistent with standard GSMR implementations. A similar approach was used in the reverse MR analysis, where genetic variants linked to brain cancer were selected as potential instruments for exploring a bidirectional relationship. To further refine the instrument set, we implemented the HEIDI‐outlier test to detect and exclude SNPs with strong pleiotropic effects, applying a conservative threshold of *p* < 0.01. The HEIDI‐outlier flag was enabled (heidi_outlier_flag = TRUE) to test for heterogeneity in effect sizes, removing SNPs with *p*_HEIDI_ < 0.01. This procedure identifies SNPs whose effect estimates on the exposure and outcome are inconsistent, helping to minimize horizontal pleiotropy. Additionally, only SNPs with *F*‐statistics greater than 10 were retained to ensure adequate instrument strength, and a minimum of three independent SNPs was required to perform GSMR.

### 2.6 Data Analysis

MR analyses were performed using the “TwoSampleMR” and “GSMR” packages in R version 4.3.1. To ensure the robustness and reliability of causal inference, we implemented a multi‐step framework. The IVW method was employed as the primary analytical approach, as it efficiently estimates causal effects under the assumption of no horizontal pleiotropy. Associations were considered statistically significant if the IVW method met the predefined significance threshold (*p* < 0.05). To enhance the credibility of our findings, we required consistent effect directionality across multiple MR approaches, including GSMR, IVW, MR‐Egger regression, and the weighted median estimator. This strategy helped to mitigate biases arising from method‐specific limitations and ensured that conclusions were not driven by a single analytical method. As an additional validation step, GSMR was applied as a confirmatory analysis, with results deemed significant only if they met the *p* < 0.05 threshold. To account for potential pleiotropic effects, we conducted Cochran's Q test on IVW estimates, where a non‐significant result indicated minimal heterogeneity. Directional pleiotropy was assessed separately using the MR‐Egger intercept test.

## 3 Results

### 3.1 The Causal Effect of Genetically Predicted *T. Gondii* Seropositivity on Brain Tumor Risk

**FIGURE 2:**
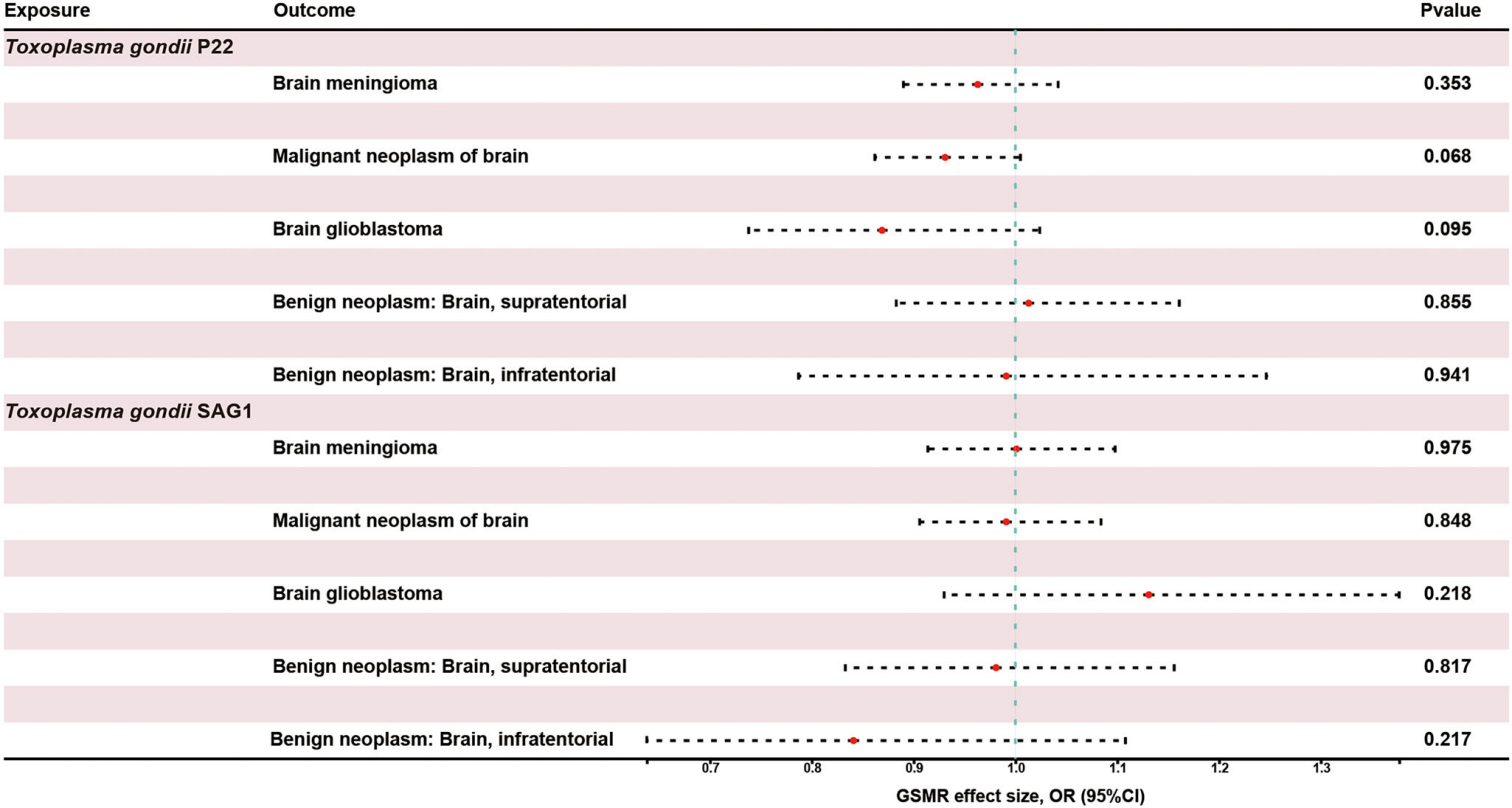
**Forest plot for the GSMR results of *T. gondii* antibody seropositivity on the risk of brain tumor. Abbreviations**: CI, confidence interval; OR, odds ratio representing the magnitude of the causal effect; *p*, *p*‐value.

To assess potential causal effects of genetically proxied *T. gondii* serological on risk of brain‐tumor subtypes, we performed two‐sample Mendelian randomization using IVW, weighted‐median, MR‐Egger, and GSMR methods. Here, we present the findings for the two antibody markers, P22 and SAG1. For genetically proxied P22 antibody levels, MR analyses did not reveal statistically significant evidence of a causal effect on the risk of brain tumor. IVW estimates did not indicate statistically significant associations between genetically proxied P22 levels and specific tumor subtypes: meningioma (OR = 0.976; 95% CI: 0.876–1.087; *p* = 0.656), supratentorial benign neoplasms (OR = 1.026; 95% CI: 0.890–1.181; *p* = 0.725), or infratentorial benign neoplasms (OR = 1.016; 95% CI: 0.776–1.330; *p* = 0.909) (**Table** **2**, **Supplementary Table S1–S2**). A suggestive inverse trend was observed for glioblastoma (OR = 0.863; 95% CI: 0.735–1.014; *p* = 0.073) and for overall malignant brain tumors (OR = 0.930; 95% CI: 0.863–1.002; *p* = 0.056), but these estimates did not reach conventional statistical significance (**Table** **2** **and** **Supplementary Table** **2**). MR analyses using the weighted median and MR‐Egger approaches produced results consistent with the primary findings, providing no evidence of significant causal effects. Similarly, GSMR results were broadly consistent with the IVW estimates and did not provide evidence for causal effects across tumor subtypes (**Figure** **2**).

**TABLE 2:** MR results of *T. gondii* antibody seropositivity on brain tumor risk.

Exposure	Outcome	Inverse variance weighted		Weighted median		MR Egger		GSMR	
		OR (95% CI)	*p*‐value	OR (95% CI)	*p*‐value	OR (95% CI)	*p‐*value	OR (95% CI)	*p*‐value
P22	Brain meningioma	0.97 6(0.876, 1.087)	0.656	0.937 (0.836, 1.05)	0.260	0.871 (0.685, 1.109)	0.278	0.963 (0.89, 1.042)	0.353
P22	Malignant neoplasm of brain	0.93 (0.863, 1.002)	0.056	0.904 (0.81, 1.009)	0.073	0.837 (0.709, 0.989)	0.052	0.931 (0.862, 1.005)	0.068
P22	Brain glioblastoma	0.863 (0.735, 1.014)	0.073	0.852 (0.683, 1.062)	0.155	0.735 (0.514, 1.051)	0.109	0.869 (0.738, 1.024)	0.095
P22	Benign neoplasm: Brain, supratentorial	1.026 (0.89, 1.181)	0.725	0.963 (0.799, 1.161)	0.691	0.952 (0.704, 1.287)	0.752	1.013 (0.883, 1.161)	0.855
P22	Benign neoplasm: Brain, infratentorial	1.016 (0.776, 1.33)	0.909	0.919 (0.66, 1.28)	0.617	1.054 (0.571, 1.946)	0.868	0.991 (0.787, 1.248)	0.941
SAG1	Brain meningioma	1.004 (0.917, 1.099)	0.926	0.992 (0.867, 1.136)	0.907	1.013 (0.855, 1.2)	0.885	1.001 (0.914, 1.098)	0.975
SAG1	Malignant neoplasm of brain	0.99 (0.906, 1.082)	0.829	0.991 (0.869, 1.13)	0.890	0.978 (0.829, 1.155)	0.798	0.991 (0.906, 1.084)	0.848
SAG1	Brain glioblastoma	1.142 (0.935, 1.394)	0.193	1.036 (0.767, 1.399)	0.817	1.054 (0.723, 1.536)	0.788	1.131 (0.93, 1.377)	0.218
SAG1	Benign neoplasm: Brain, supratentorial	0.983 (0.836, 1.155)	0.830	0.951 (0.761, 1.188)	0.656	0.952 (0.704, 1.287)	0.752	0.981 (0.833, 1.156)	0.817
SAG1	Benign neoplasm: Brain, infratentorial	0.831 (0.633, 1.091)	0.183	0.833 (0.558, 1.245)	0.373	0.794 (0.477, 1.32)	0.380	0.841 (0.638, 1.108)	0.217

**Abbreviations**: CI, confidence interval; MR, Mendelian randomization; OR, odds ratio; *T. gondii*, *Toxoplasma gondii*.

A similar pattern was observed for SAG1 antibody levels, where no statistically significant causal associations with the risk of brain tumors were detected. IVW estimates for SAG1 were close to null and did not reach statistical significance for meningioma (OR = 1.004; 95% CI: 0.917–1.099; *p* = 0.926), glioblastoma (OR = 1.142; 95% CI: 0.935–1.394; *p* = 0.193), supratentorial benign neoplasms (OR = 0.983; 95% CI: 0.836–1.155; *p* = 0.830), infratentorial benign neoplasms (OR = 0.831; 95% CI: 0.633–1.091; *p* = 0.183), or for overall malignant brain tumors (OR = 0.990; 95% CI: 0.906–1.082; *p* = 0.829) (**Table** **2** **and** **Supplementary Tables S1–S2**). Weighted‐median and MR‐Egger estimates yielded consistent effect estimates, and GSMR likewise did not provide evidence for causal effects across tumor subtypes (**Figure** **2** **and** **Table** **2**). In addition, MR‐Egger intercept tests were not statistically significant (*p* > 0.05), providing no evidence of directional pleiotropy (**Supplementary Table S3**). Heterogeneity analysis using Cochran's *Q*‐test revealed no significant heterogeneity for most associations (**Supplementary Table S4**). However, heterogeneity was detected in the relationship between *T. gondii* P22 antibody levels and brain meningioma (*Q* = 36.82, df = 18, *p* = 0.005). Collectively, these MR analyses did not provide evidence that genetically predicted *T. gondii* seropositivity has a causal effect on risk of brain tumors, across both malignant and benign subtypes.

### 3.2 The Causal Effect of Brain Tumors on *T. gondii* Seropositivity

**FIGURE 3:**
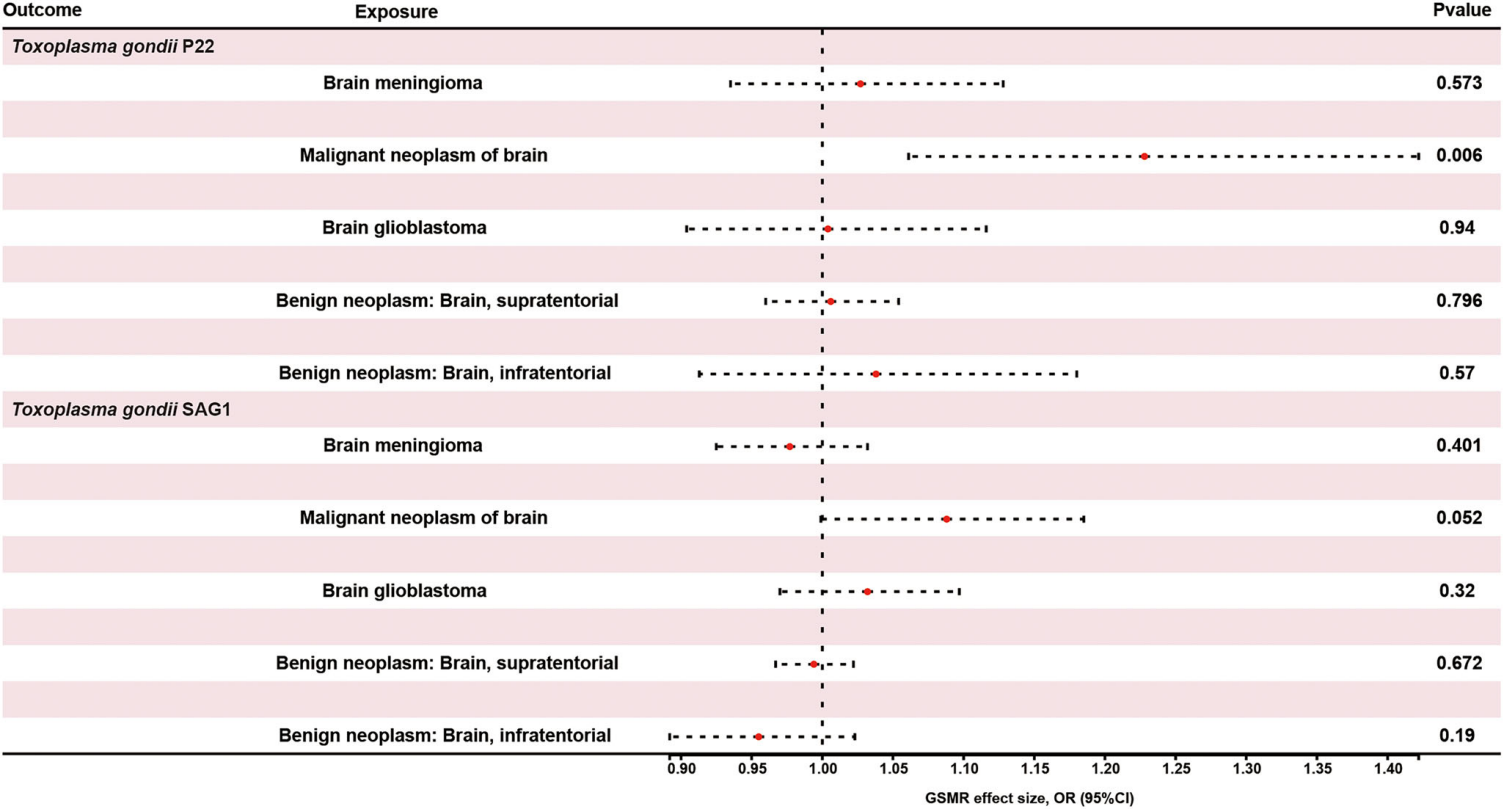
**Forest plot for the GSMR results of brain tumors on seropositivity for *T. gondii* antibody. Abbreviations**: CI, confidence interval; OR, odds ratio representing the magnitude of the causal effect; *p*, *p*‐value.

In reverse MR analyses, genetically predicted liability to malignant brain tumors was associated with higher genetically predicted *T. gondii* seropositivity, particularly for the P22 antibody (**Table** **3** **;** **Supplementary Tables S5–S6**). Specifically, IVW analysis estimated an OR of 1.234 (95% CI: 1.069–1.425; *p* = 0.0042) for P22, which remained statistically significant after Bonferroni correction. Weighted‐median (OR = 1.157; 95% CI: 0.964–1.389; *p* = 0.117) and MR‐Egger (OR = 1.245; 95% CI: 0.852–1.820; *p* = 0.291) yielded directionally consistent but non‐significant estimates. GSMR produced a comparable OR of 1.234 (95% CI: 1.069–1.425; *p* = 0.0058), confirming consistency across methods (**Figure** **3** **and** **Table** **3**). For SAG1, IVW analysis indicated a borderline positive association (OR = 1.095; 95% CI: 1.001–1.197; *p* = 0.048), with weighted‐median (OR = 1.114; 95% CI: 0.995–1.246; *p* = 0.061) and MR‐Egger (OR = 0.986; 95% CI: 0.778–1.251; *p* = 0.912) showing directionally consistent but not statistically significant effects. GSMR yielded a similar borderline OR of 1.095 (95% CI: 1.001–1.197; *p* = 0.052). No significant associations were observed between genetically predicted liability to meningioma, glioblastoma, or benign brain neoplasms and genetically predicted P22 or SAG1 seropositivity, with all ORs close to 1 and *p*‐values > 0.05. Sensitivity analyses did not indicate substantial heterogeneity or directional pleiotropy (**Supplementary Tables S7–S8**), and effect estimates were generally consistent across MR methods. These findings suggest that genetic predisposition to malignant brain tumors may increase genetically predicted *T. gondii* seropositivity, although causal mechanisms cannot be directly inferred.

**TABLE 3:** The reverse MR results of brain tumor on *T. gondii* antibody seropositivity.

Exposure	Outcome	Inverse variance weighted		Weighted median		MR Egger		GSMR	
		OR (95% CI)	*p*‐value	OR (95% CI)	*p*‐value	OR (95% CI)	*p*‐value	OR (95% CI)	*p*‐value
Brain meningioma	P22	1.019 (0.914, 1.135)	0.739	0.978 (0.844, 1.134)	0.772	0.947 (0.737, 1.217)	0.677	1.027 (0.935, 1.128)	0.573
Malignant neoplasm of brain	P22	1.234 (1.069, 1.425)	**0.004**	1.157 (0.964, 1.389)	0.117	1.245 (0.852, 1.82)	0.291	1.228 (1.061, 1.422)	**0.006**
Brain glioblastoma	P22	1.004 (0.904, 1.114)	0.945	1.022 (0.901, 1.158)	0.738	0.77 (0.443, 1.337)	0.421	1.004 (0.904, 1.116)	0.940
Benign neoplasm: Brain, supratentorial	P22	1.046 (0.912, 1.201)	0.520	1.032 (0.894, 1.191)	0.666	1.078 (0.772, 1.505)	0.679	1.006 (0.96, 1.054)	0.796
Benign neoplasm: Brain, infratentorial	P22	1.049 (0.862, 1.276)	0.634	1.021 (0.872, 1.195)	0.795	0.882 (0.519, 1.499)	0.674	1.038 (0.913, 1.18)	0.570
Brain meningioma	SAG1	1.009 (0.947, 1.076)	0.775	1.006 (0.923, 1.097)	0.891	1.017 (0.881, 1.175)	0.819	0.977 (0.925, 1.032)	0.401
Malignant neoplasm of brain	SAG1	1.095 (1.001, 1.197)	**0.048**	1.114 (0.995, 1.246)	0.061	0.986 (0.778, 1.251)	0.912	1.088 (0.999, 1.185)	0.052
Brain glioblastoma	SAG1	1.033 (0.972, 1.097)	0.299	1.034 (0.956, 1.118)	0.406	1.152 (0.839, 1.58)	0.446	1.032 (0.97, 1.097)	0.320
Benign neoplasm: Brain, supratentorial	SAG1	1.011 (0.923, 1.107)	0.816	1.066 (0.977, 1.163)	0.149	0.964 (0.774, 1.201)	0.757	0.994 (0.967, 1.022)	0.672
Benign neoplasm: Brain, infratentorial	SAG1	0.951 (0.879, 1.03)	0.218	0.967 (0.888, 1.054)	0.443	1.054 (0.863, 1.286)	0.643	0.955 (0.892, 1.023)	0.190

**Abbreviations**: CI, confidence interval; MR, Mendelian randomization; OR, odds ratio; *T. gondii*, *Toxoplasma gondii*.

## 4 Discussion

To our knowledge, this is the first study to systematically investigate the causal relationship between genetically predicted *T. gondii* seropositivity and brain tumor risk using MR. Our analyses yielded two key findings: first, we found no evidence supporting a causal effect of *T. gondii* seropositivity on the risk of developing brain tumors, including malignant and benign subtypes. Second, reverse MR analysis revealed a significant positive association between malignant brain tumors and an increased risk of *T. gondii* infection, suggesting that brain malignancies may influence host susceptibility to this parasitic infection.

*T. gondii*, an obligate intracellular parasite capable of infecting a wide range of warm‐blooded animals, including humans, exhibits a strong tropism for the central nervous system and has been implicated in various neurological and neuropsychiatric disorders, such as Alzheimer's disease, schizophrenia, bipolar disorder, and attention deficit hyperactivity disorder (Torrey 2024; Graham et al. 2021). Once inside the central nervous system, *T. gondii* can establish a chronic latent infection by forming tissue cysts that persist within neurons and glial cells. The infection process involves key antigenic components, including P22 and SAG1, which play distinct roles in host invasion and immune response (Odaert et al. 1996). P22 is a major surface antigen expressed during the tachyzoite stage, crucial for host cell adhesion and invasion, and its corresponding antibody serves as an early marker of active infection. Similarly, SAG1, another well‐characterized tachyzoite antigen, is highly expressed and essential for parasite entry into host cells, making its antibody a valuable serological marker for early‐stage infection diagnosis. However, contrasting evidence indicates that chronic infection may contribute to behavioral alterations and serve as a risk factor for neurodegenerative diseases such as Alzheimer's disease (Galeh et al. 2023). The mechanisms underlying these divergent effects remain unclear but are likely mediated through a complex interplay of neuroinflammation, immune response modulation, and direct parasite‐host cell interactions.

Epidemiological studies have suggested a potential link between *T. gondii* infection and brain tumor risk, with some reports indicating higher seroprevalence of *T. gondii* antibodies in brain cancer patients across different ancestries (Thomas et al. 2012; Vittecoq et al. 2012; Cong et al. 2015; Jung et al. 2016). Given the parasite's ability to establish a chronic infection in neural tissue and modulate host immune responses, a plausible hypothesis is that *T. gondii* could contribute to tumorigenesis through inflammation, immune evasion, or direct cellular effects (Dzutsev et al. 2015; Greten and Grivennikov 2019; Kaneda et al. 2012). Consistent with previous studies, our reverse MR analysis reveals that brain malignancies increase the risk of *T. gondii* infection. Tumors can alter immune responses or change the brain's microenvironment, which may make it easier for *T. gondii* to infect the brain. Tumors can profoundly alter immune responses and reshape the brain microenvironment, potentially facilitating parasite persistence or reactivation. Importantly, this association should not be interpreted as evidence of a direct causal effect of brain tumors on incident *T. gondii* infection, but rather as reflecting a complex interplay between tumor‐related immune dysfunction and host serological response. Moreover, this association may be influenced by the combined effects of tumor‐induced immunosuppression and common treatment modalities, including chemotherapy, corticosteroids, and radiotherapy, all of which can impair cell‐mediated immunity and alter antibody dynamics (Hughes et al. 2005; Grossman et al. 2011). It is also important to note that seropositivity reflects antibody‐mediated immune response rather than active infection per se, and elevated antibody levels may indicate latent infection reactivation or altered immune surveillance in the context of malignancy.

As our analysis is based on genetic summary data, we are unable to disentangle intrinsic tumor biology from therapy‐induced immunosuppression, highlighting the need for future cohort studies with detailed clinical and treatment data to clarify these mechanisms. Accordingly, these findings should be regarded as hypothesis‐generating rather than definitive evidence of causality. Future cohort studies integrating longitudinal clinical data, treatment information, and immune profiling will be essential to clarify the temporal and biological mechanisms linking brain tumors and *T. gondii* serological responses. Taken together, these findings highlight the importance of considering both tumor‐related and treatment‐related factors in assessing *T. gondii* infection risk. For clinicians, this means that when treating patients with brain tumors, the potential risk of *T. gondii* infection, especially in those with weakened immune systems, should be carefully considered. Regular screening and early intervention could help manage these risks. For researchers, these findings open new pathways to explore how immune changes in brain tumors may influence *T. gondii* infection and vice versa, potentially guiding more personalized treatment approaches for both conditions.

We also observed evidence of heterogeneity in the association between genetically predicted P22 seropositivity and meningioma risk, as indicated by a significant Cochran's Q statistic. This finding suggests variability in SNP‐specific causal estimates, which may arise from several features of serological traits and MR instrumentation. In particular, antibody seropositivity reflects a complex, polygenic immune response rather than a single biological process, and individual genetic instruments may influence infection susceptibility, antibody production, or immune regulation through partially distinct pathways. Such heterogeneity in instrument–exposure mechanisms can contribute to between‐SNP variability in causal estimates. In addition, heterogeneity may reflect differences in instrument strength or the presence of balanced horizontal pleiotropy, which can increase variability without inducing systematic bias. Importantly, heterogeneity alone does not invalidate MR results, especially in the absence of directional pleiotropy. In our analyses, the MR‐Egger intercept test did not provide evidence of unbalanced horizontal pleiotropy, and causal estimates derived from multiple complementary MR methods, including inverse‐variance weighted, weighted median, MR‐Egger, and GSMR approaches, were directionally consistent and close to the null. Taken together, these observations suggest that the detected heterogeneity is unlikely to materially bias the overall conclusions. Nevertheless, this finding underscores the complexity of antibody‐mediated immune response phenotypes and highlights the importance of cautious interpretation, particularly in subtype‐specific analyses such as meningioma, where the number of available instruments and outcome events is relatively limited.

Interestingly, our findings provide no evidence supporting a causal relationship between *T. gondii* infection as an exposure and brain tumors as an outcome. This suggests that the previously observed epidemiological associations between *T. gondii* infection and brain cancer risk may be confounded by unmeasured factors or may reflect reverse causality, rather than a direct causal effect of the parasite. Several biological explanations could account for this null result. First, *T. gondii* infection is known to activate the host's Th1‐type immune response, which enhances T‐cell infiltration into tumors. This mechanism may, in some cases, exert an anti‐tumor effect (Nguyen et al. 2024). In addition to its acute immune‐stimulating properties, chronic *T. gondii* infection has also been shown to possess anti‐tumor activity. Studies have demonstrated that mice infected with *T. gondii* exhibit elevated serum levels of TNF‐α and IFN‐γ, two key pro‐inflammatory cytokines known to play critical roles in tumor suppression (Li et al. 2021). These cytokines may contribute to an immunological environment that is unfavorable for tumor progression, potentially counteracting any tumor‐promoting effects associated with the parasite. This suggests that *T. gondii* infection may have an immunomodulatory role that, under certain conditions, suppresses rather than promotes tumor growth. Second, our MR approach reflects lifetime exposure to *T. gondii* rather than acute infections, implying that persistent antibody levels do not substantially contribute to tumorigenesis. These factors collectively reinforce the robustness of our null findings, suggesting that while *T. gondii* infection can influence the immune microenvironment, it does not play a direct causal role in brain tumor development.

In our study, the category “Malignant neoplasm of brain” encompasses multiple primary malignant subtypes, including glioblastomas, high‐grade astrocytomas, and other rarer malignancies. Analyzing this aggregated group, we observed a significant positive association with *T. gondii* serological response, suggesting that genetic liability to malignant brain tumors may influence host susceptibility or immune response to infection. However, when glioblastoma was examined separately, no statistically significant association was detected. This discrepancy may reflect both statistical and biological factors. Glioblastoma cases are fewer in number, reducing power to detect modest effects. Biologically, glioblastoma exhibits a highly aggressive and immunosuppressive microenvironment, with abundant myeloid‐derived suppressor cells, regulatory T cells, and immune checkpoint molecules, which may limit the parasite's ability to establish infection or the detectability of genetic associations (Quail and Joyce 2017; Omuro and DeAngelis 2013). Together, these findings suggest that the association observed for the aggregated malignant brain tumor category may arise from shared genetic or immunological pathways across subtypes that influence host‐parasite interactions, whereas glioblastoma's distinct biology may attenuate or mask these effects. Future studies with larger sample sizes and stratified analyses by molecular and histological subtypes will be essential to clarify subtype‐specific mechanisms linking brain tumor biology and host immune response to *T. gondii*.

However, several limitations should be acknowledged. First, our study relied on genetically predicted serological antibody levels (P22 and SAG1) as proxies for *T. gondii* exposure. While these markers are well‐established indicators of immune response, they do not distinguish between latent and active infection, nor do they quantify parasite load. Therefore, our findings should be interpreted as reflecting the genetic liability to *T. gondii* seropositivity rather than actual infection status, and further studies using more precise infection metrics are warranted. Second, our analyses were restricted to individuals of European ancestry, and the results may not generalize to populations with different genetic backgrounds or *T. gondii* exposure rates. Finally, the observed association between genetic liability to malignant brain tumors and genetically predicted *T. gondii* seropositivity warrants further investigation, particularly regarding the influence of specific tumor subtypes and treatment regimens. Furthermore, these findings should be interpreted with caution as no correction for multiple testing was performed.

## 5 Conclusion

Our study provides robust genetic evidence that genetically predicted *T. gondii* seropositivity is not causally associated with an increased risk of brain tumors. Conversely, we show that genetic liability to malignant brain tumors is associated with higher genetically predicted *T. gondii* seropositivity. These findings suggest a potential genetic interplay between brain tumor susceptibility and immune response to *T. gondii* exposure, warranting further research to explore the underlying biological mechanisms and possible clinical implications.

## Author Contributions

P. S., G. W., and Z. L. contributed to investigation, methodology, and drafting the initial manuscript. B. D., G. Z., J. Z. Y. W., and Z. C. revised and refined the manuscript. Z. C. conceived and supervised the study.

## Funding

This research was funded by the Henan Province Medical Science and Technology Research Plan Joint Construction Project (Grant No. LHGJ20210897).

## Ethics Statement

The data used in this study were derived from publicly accessible databases, and ethical approval for the original data collection was granted by the relevant ethical review boards.

## Consent

All authors have provided explicit consent for the publication of this work.

## Conflicts of Interest

The authors declare no conflicts of interest.

## Supporting information




**Supplementary Materials**: brb371239‐sup‐0001‐Tables.pdf

## Data Availability

The datasets utilized in this study are publicly available. For further details or inquiries, please contact the corresponding authors.
