# Supplementary material for: Exploring the Causal Links Between Toxoplasma gondii Infection and Risk of Brain Tumors: A Bidirectional Mendelian Randomization Analysis
Source: Brain Behav. 2026 Jan 29;16(2):e71239. doi: 10.1002/brb3.71239 (PMC12856237; doi:10.1002/brb3.71239)
Supplement: Supplementary file 1 — Supplementary Materials: brb371239‐sup‐0001‐Tables.pdf [file BRB3-16-e71239-s001.pdf]

Supplementary Table 1. Instrumental variables associated with T. gondii antibody seropositivity used in the MR analyses

ID	Trait	SNP	effect_allele	other_allele	effect_allele_frequency	beta	standard_error	p_value	chromosome	base_pair_location
GCST90006926	P22	rs111795967	A	G	0.0250784	0.586913	0.123233	1.9108E-06	1	153847622
GCST90006926	P22	rs56226606	A	C	0.144028	0.267659	0.0552033	1.2433E-06	1	219204781
GCST90006926	P22	rs74963268	T	C	0.113016	0.287447	0.0612318	2.6739E-06	1	231367963
GCST90006926	P22	rs145278907	T	C	0.0232372	0.603845	0.126803	1.916E-06	3	84145105
GCST90006926	P22	rs187264632	G	C	0.00510605	1.36057	0.271981	5.661E-07	4	12781350
GCST90006926	P22	rs114155328	A	G	0.0956113	0.301327	0.0653772	4.045E-06	4	83641161
GCST90006926	P22	rs114521655	T	C	0.0477308	0.442299	0.0912881	1.2655E-06	4	96303295
GCST90006926	P22	rs147859115	T	C	0.00746269	1.19908	0.225226	1.0158E-07	6	104576285
GCST90006926	P22	rs35572225	TG	T	0.159671	0.272634	0.0541872	4.8707E-07	7	123509744
GCST90006926	P22	rs139859109	G	A	0.00801688	1.18923	0.225397	1.3191E-07	8	240804
GCST90006926	P22	rs77965248	C	A	0.0169158	0.716561	0.151518	2.2538E-06	10	113097112
GCST90006926	P22	rs72930159	T	C	0.0479345	-0.41051	0.0894936	4.4964E-06	11	63258123
GCST90006926	P22	rs17811389	T	C	0.0117279	0.835621	0.180498	3.665E-06	11	75946352
GCST90006926	P22	rs143874551	T	C	0.00780031	1.12703	0.21977	2.9253E-07	11	87933554
GCST90006926	P22	rs112698198	A	G	0.03362	0.492169	0.107769	4.9502E-06	12	72767413
GCST90006926	P22	rs74920744	A	T	0.0132502	0.807272	0.169736	1.9743E-06	13	73857735
GCST90006926	P22	rs144983967	A	T	0.0109718	0.869063	0.186672	3.2308E-06	15	66957892
GCST90006926	P22	rs112090875	T	C	0.0145784	0.750752	0.15859	2.2023E-06	17	1480409
GCST90006926	P22	rs78755026	A	G	0.022993	0.62025	0.130156	1.8844E-06	17	9323684
GCST90006926	P22	rs4121993	C	T	0.797308	0.257291	0.0485765	1.1797E-07	18	49921872
GCST90006926	P22	rs193166830	T	A	0.00516283	1.31367	0.272177	1.3893E-06	21	46153278
GCST90006927	SAG1	rs55710143	G	C	0.0381648	0.310501	0.0611609	3.8387E-07	1	5090490
GCST90006927	SAG1	rs34863121	T	C	0.0127438	0.472729	0.101327	3.0802E-06	1	109823458
GCST90006927	SAG1	rs76488573	A	G	0.0492296	0.25606	0.0529271	1.3117E-06	2	3080866
GCST90006927	SAG1	rs80071386	A	T	0.0111344	0.51271	0.104871	1.0136E-06	2	118919931
GCST90006927	SAG1	rs540392	A	C	0.536792	-0.10717	0.0229134	2.9082E-06	3	10488641
GCST90006927	SAG1	rs6550258	G	C	0.767666	-0.12996	0.0275392	2.3678E-06	3	34241097
GCST90006927	SAG1	rs4342072	C	G	0.978272	-0.36757	0.0778343	2.3295E-06	3	80480149
GCST90006927	SAG1	rs823970	C	T	0.529536	-0.10521	0.0225824	3.1792E-06	3	135083504
GCST90006927	SAG1	rs112511265	C	T	0.0792066	0.193586	0.0418936	3.8209E-06	4	166035678
GCST90006927	SAG1	rs56003285	G	A	0.00681342	0.632533	0.138279	4.7774E-06	4	184233006
GCST90006927	SAG1	rs148929820	A	G	0.0254193	0.450908	0.0724204	4.7768E-10	5	120890753
GCST90006927	SAG1	rs7736504	A	G	0.00611501	0.699368	0.145303	1.4856E-06	5	170210490
GCST90006927	SAG1	rs34697429	T	A	0.0351812	0.328346	0.0619073	1.134E-07	6	32588432
GCST90006927	SAG1	rs72899128	T	G	0.0201255	0.379265	0.0814509	3.2184E-06	6	79445474
GCST90006927	SAG1	rs143797072	G	A	0.0540904	0.238872	0.0508951	2.6868E-06	6	117997633
GCST90006927	SAG1	rs117642724	G	C	0.0272655	0.325377	0.069376	2.7314E-06	8	13421695
GCST90006927	SAG1	rs149988186	A	T	0.0140845	0.437275	0.0957581	4.9606E-06	8	66673257
GCST90006927	SAG1	rs34970068	G	T	0.0120545	0.522749	0.10446	5.6071E-07	8	100843712
GCST90006927	SAG1	rs146240117	G	T	0.00561651	0.70811	0.151863	3.1189E-06	8	120982959
GCST90006927	SAG1	rs11506229	G	C	0.0311198	0.321683	0.0641413	5.2973E-07	9	23094037
GCST90006927	SAG1	rs10869437	T	G	0.121833	0.163361	0.033965	1.5117E-06	9	77314098
GCST90006927	SAG1	rs75136602	A	G	0.0798474	0.198427	0.041989	2.2933E-06	9	93969277
GCST90006927	SAG1	rs2279204	C	T	0.0566701	0.261326	0.0488783	8.969E-08	10	3170838
GCST90006927	SAG1	rs2268356	T	C	0.515001	-0.11216	0.0230945	1.1948E-06	10	124265326
GCST90006927	SAG1	rs17711616	G	A	0.150013	0.149407	0.0313889	1.9371E-06	11	109651529
GCST90006927	SAG1	rs3847677	G	T	0.71451	0.120664	0.0248825	1.2388E-06	12	28846917
GCST90006927	SAG1	rs2093705	C	T	0.580544	-0.11155	0.0231893	1.5049E-06	13	43265382
GCST90006927	SAG1	rs77799120	C	T	0.00666489	0.653441	0.140986	3.5728E-06	13	89566445
GCST90006927	SAG1	rs45508391	A	G	0.0539332	-0.24579	0.0500125	8.9014E-07	14	102474751
GCST90006927	SAG1	rs149699011	T	G	0.00939457	0.537988	0.117824	4.9706E-06	15	77497182
GCST90006927	SAG1	rs12930749	G	A	0.163786	0.156389	0.0304072	2.7016E-07	16	27727779
GCST90006927	SAG1	rs181020477	T	G	0.00521648	0.799392	0.157308	3.7405E-07	17	13158618
GCST90006927	SAG1	rs11881343	T	A	0.0178522	0.463093	0.0866402	9.0411E-08	19	15301857
GCST90006927	SAG1	rs77658816	A	G	0.100366	-0.17476	0.0374524	3.0693E-06	19	41795940
GCST90006927	SAG1	rs117000365	T	C	0.0114883	0.502544	0.106786	2.5254E-06	21	33745279
GCST90006927	SAG1	rs139610237	C	G	0.0162797	0.437745	0.0911308	1.5593E-06	22	33867744

IV, instrumental variable; MR, mendelian randomization; SNP, single nucleotide polymorphism; *T. gondii, Toxoplasma gondii*

Supplementary Table 2. Mendelian randomization estimates for the causal effect of T. gondii antibody seropositivity on brain tumor risk.

exposure	outcome	method	nsnp	pval	or	or_lci95	or_uci95
P22	Brain meningioma	MR Egger	19	0.278217446	-0.378484691	0.684898456	1.108709092
	Brain meningioma	Weighted median	19	0.259963027	-0.179487143	0.835698696	1.049610521
	Brain meningioma	Inverse variance weighted	19	0.656298939	-0.13266133	0.875761636	1.08715672
	Brain meningioma	Simple mode	19	0.337473701	-0.246870629	0.781241757	1.085111983
	Brain meningioma	Weighted mode	19	0.311402903	-0.222720688	0.800338362	1.070525325
SAG1	Brain meningioma	MR Egger	35	0.884836158	-0.15685101	0.854831411	1.199725784
	Brain meningioma	Weighted median	35	0.906748039	-0.143241765	0.866544544	1.135513978
	Brain meningioma	Inverse variance weighted	35	0.926131086	-0.086184373	0.917425067	1.099375997
	Brain meningioma	Simple mode	35	0.486812791	-0.31783205	0.727724999	1.161852919
	Brain meningioma	Weighted mode	35	0.824964613	-0.189141724	0.827669198	1.162436265
P22	Malignant neoplasm of brain	MR Egger	19	0.05154548	0.837407303	0.709264424	0.98870177
	Malignant neoplasm of brain	Weighted median	19	0.072755145	0.904399042	0.810385797	1.00931881
	Malignant neoplasm of brain	Inverse variance weighted	19	0.055811001	0.929651756	0.862688317	1.00181302
	Malignant neoplasm of brain	Simple mode	19	0.163122372	0.876442202	0.733704973	1.046947972
	Malignant neoplasm of brain	Weighted mode	19	0.164368771	0.884004545	0.748266094	1.044366493
SAG1	Malignant neoplasm of brain	MR Egger	35	0.798275353	0.978425718	0.828842108	1.155005128
	Malignant neoplasm of brain	Weighted median	35	0.889763705	0.990768288	0.868986799	1.12961647
	Malignant neoplasm of brain	Inverse variance weighted	35	0.828564496	0.990237014	0.906093249	1.082194735
	Malignant neoplasm of brain	Simple mode	35	0.975251201	0.996156139	0.782402278	1.268307981
	Malignant neoplasm of brain	Weighted mode	35	0.844716176	1.017880591	0.853613523	1.213758767
P22	Brain glioblastoma	MR Egger	19	0.109490216	0.73478304	0.513850037	1.050707554
	Brain glioblastoma	Weighted median	19	0.154685172	0.851978786	0.683305936	1.062288229
	Brain glioblastoma	Inverse variance weighted	19	0.073454277	0.863078358	0.734559402	1.014083067
	Brain glioblastoma	Simple mode	19	0.677085472	0.924974897	0.644621484	1.327257283
	Brain glioblastoma	Weighted mode	19	0.221443689	0.798590513	0.563865706	1.131026058
SAG1	Brain glioblastoma	MR Egger	35	0.787851892	1.053600459	0.722524913	1.536381524
	Brain glioblastoma	Weighted median	35	0.81732393	1.035999961	0.767411058	1.398593241
	Brain glioblastoma	Inverse variance weighted	35	0.192762345	1.14170661	0.935283545	1.393688566
	Brain glioblastoma	Simple mode	35	0.974617653	1.009871171	0.553863255	1.841320529
	Brain glioblastoma	Weighted mode	35	0.932257913	1.021335994	0.629972151	1.655830676
P22	Benign neoplasm: Brain, supratentorial	MR Egger	19	0.743761757	0.948403014	0.693857658	1.296329682
	Benign neoplasm: Brain, supratentorial	Weighted median	19	0.690694679	0.962788116	0.79874453	1.160522447
	Benign neoplasm: Brain, supratentorial	Inverse variance weighted	19	0.7251072	1.025694461	0.890441033	1.181492192
	Benign neoplasm: Brain, supratentorial	Simple mode	19	0.86630265	0.973597877	0.71616732	1.323563362
	Benign neoplasm: Brain, supratentorial	Weighted mode	19	0.557562222	0.926985997	0.722898177	1.188691667
SAG1	Benign neoplasm: Brain, supratentorial	MR Egger	35	0.752191946	0.952192461	0.704298473	1.287338418
	Benign neoplasm: Brain, supratentorial	Weighted median	35	0.655928793	0.950695347	0.76110075	1.187519053
	Benign neoplasm: Brain, supratentorial	Inverse variance weighted	35	0.830275882	0.982500306	0.836031617	1.154629599
	Benign neoplasm: Brain, supratentorial	Simple mode	35	0.708852296	0.920173568	0.59676494	1.418849096
	Benign neoplasm: Brain, supratentorial	Weighted mode	35	0.547652402	0.896035461	0.628742412	1.276961012
P22	Benign neoplasm: Brain, infratentorial	MR Egger	19	0.867723985	1.05429996	0.571176932	1.946066698
	Benign neoplasm: Brain, infratentorial	Weighted median	19	0.617261654	0.919036669	0.659965697	1.279806513
	Benign neoplasm: Brain, infratentorial	Inverse variance weighted	19	0.90886837	1.015859526	0.775927341	1.329983519
	Benign neoplasm: Brain, infratentorial	Simple mode	19	0.747876759	0.912751447	0.527573127	1.579146401
	Benign neoplasm: Brain, infratentorial	Weighted mode	19	0.77468549	0.926582462	0.554014953	1.549696547
SAG1	Benign neoplasm: Brain, infratentorial	MR Egger	35	0.379520294	0.793507725	0.477002078	1.320024668
	Benign neoplasm: Brain, infratentorial	Weighted median	35	0.373182583	0.833258572	0.557725747	1.244912669
	Benign neoplasm: Brain, infratentorial	Inverse variance weighted	35	0.182630915	0.831116457	0.63315283	1.090976037
	Benign neoplasm: Brain, infratentorial	Simple mode	35	0.433699291	0.745244436	0.360061132	1.542486036
	Benign neoplasm: Brain, infratentorial	Weighted mode	35	0.877392387	0.957990089	0.557619093	1.645827808

MR, mendelian randomization; *T. gondii, Toxoplasma gondii*; nsnp, number of single-nucleotide polymorphisms (SNPs) used in the analysis; pval, p-value indicating the statistical significance of the causal estimate; or, odds ratio representing the magnitude of the causal effect; or_lci95, lower bound of the 95% confidence interval for the odds ratio; or_uci95, upper bound of the 95% confidence interval for the odds ratio

Supplementary Table 3. Pleiotropy assessment of T. gondii antibody seropositivity on brain tumor risk using the MR-Egger intercept test.

exposure	outcome	egger_intercept	se	pval
P22	Brain meningioma	0.054623903	0.053054684	0.317635235
SAG1	Brain meningioma	-0.002160982	0.018941045	0.909857853
P22	Malignant neoplasm of brain	0.050487384	0.036557124	0.185148291
SAG1	Malignant neoplasm of brain	0.003114549	0.018559699	0.867755035
P22	Brain glioblastoma	0.077844684	0.078785992	0.336982334
SAG1	Brain glioblastoma	0.02082029	0.042163487	0.62471889
P22	Benign neoplasm: Brain, supratentorial	0.038671633	0.069821833	0.58688562
SAG1	Benign neoplasm: Brain, supratentorial	0.008137579	0.033751053	0.810964829
P22	Benign neoplasm: Brain, infratentorial	-0.01823616	0.136955224	0.895634939
SAG1	Benign neoplasm: Brain, infratentorial	0.01199678	0.056853747	0.834176153

T. gondii, Toxoplasma gondii; se, Standard error; pval, p-value

Supplementary Table 4. Heterogeneity assessment of T. gondii antibody seropositivity on brain tumor risk using Cochran's Q test

exposure	outcome	Q	Q_df	Q_pval
P22	Brain meningioma	36.82227133	18	0.005527009
SAG1	Brain meningioma	24.23007153	34	0.892343593
P22	Malignant neoplasm of brain	15.05761733	18	0.658007142
SAG1	Malignant neoplasm of brain	20.29276115	34	0.969640373
P22	Brain glioblastoma	11.97427805	18	0.848562638
SAG1	Brain glioblastoma	36.83831362	34	0.338898617
P22	Benign neoplasm: Brain, supratentorial	20.11931685	18	0.326142429
SAG1	Benign neoplasm: Brain, supratentorial	22.98879124	34	0.923854844
P22	Benign neoplasm: Brain, infratentorial	26.21014693	18	0.095049141
SAG1	Benign neoplasm: Brain, infratentorial	28.25918365	34	0.744598648

T. gondii, Toxoplasma gondii

Supplementary Table 5. Instrumental variables associated with each brain tumor subtype used in the reverse MR analyses.

Trait	SNP	effect_allele	other_allele	effect_allele frequency	beta	standard_error	p_value	chromosome	base_pair_location
Brain meningioma	rs146251630	C	T	0.0124856	0.605488	0.121602	6.38308E-07	1	33569884
Brain meningioma	rs13062210	T	A	0.314468	-0.191213	0.0364652	1.57387E-07	3	169831985
Brain meningioma	rs9843144	A	G	0.0105928	0.623948	0.132026	2.29066E-06	3	191717952
Brain meningioma	rs13119772	A	G	0.337634	-0.167616	0.0355022	2.34342E-06	4	113825053
Brain meningioma	rs112013179	G	C	0.120609	0.222358	0.0481896	3.94566E-06	4	170152818
Brain meningioma	rs112691709	C	G	0.0105267	-1.01011	0.218831	3.91336E-06	7	143040327
Brain meningioma	rs12704053	G	C	0.661846	0.233702	0.0357906	6.59022E-11	7	148905450
Brain meningioma	rs62523388	A	C	0.262662	-0.176812	0.0387048	4.91881E-06	8	143160497
Brain meningioma	rs12770228	A	G	0.327561	0.166305	0.0346717	1.6141E-06	10	21494705
Brain meningioma	rs58742727	G	A	0.945191	-0.424598	0.0650637	6.76083E-11	10	104071252
Brain meningioma	rs276209	G	T	0.0300813	0.446038	0.0852391	1.66978E-07	10	104312769
Brain meningioma	rs1856004	G	A	0.0673897	0.29484	0.060916	1.29772E-06	10	106821779
Brain meningioma	rs72878024	A	G	0.0778384	-0.480114	0.0701333	7.60852E-12	11	199492
Brain meningioma	rs7925931	G	A	0.968719	-0.39648	0.0848891	3.00379E-06	11	59389777
Brain meningioma	rs10735352	G	C	0.373142	0.165421	0.0337786	9.72076E-07	12	97266984
Brain meningioma	rs9544074	G	A	0.394506	-0.175933	0.0341264	2.53175E-07	13	75900302
Brain meningioma	rs117282189	G	A	0.0320874	0.390226	0.0844734	3.84636E-06	16	69507018
Brain meningioma	rs73961603	C	T	0.131852	-0.268445	0.0519228	2.34002E-07	18	58554276
Brain meningioma	rs134481	T	C	0.820293	-0.20021	0.0413761	1.30629E-06	22	28325064
Brain meningioma	rs62235753	T	C	0.0109445	0.672996	0.130106	2.3077E-07	22	28876649
Malignant neoplasm of brain	rs4694639	T	C	0.564235	-0.15104	0.0326035	3.61044E-06	4	73833337
Malignant neoplasm of brain	rs2242652	A	G	0.230059	0.293733	0.0363665	6.6359E-16	5	1279913
Malignant neoplasm of brain	rs76928645	T	C	0.116207	0.247762	0.0474627	1.78785E-07	7	54873635
Malignant neoplasm of brain	rs55705857	G	A	0.0965465	0.475377	0.0481123	5.05475E-23	8	129633446
Malignant neoplasm of brain	rs3217992	T	C	0.386121	-0.19758	0.0337531	4.80817E-09	9	22003224
Malignant neoplasm of brain	rs77728719	A	G	0.0211071	0.465408	0.0979535	2.02102E-06	10	27947047
Malignant neoplasm of brain	rs17670780	G	T	0.0787179	0.259643	0.0560381	3.5984E-06	12	16643993
Malignant neoplasm of brain	rs17459793	T	C	0.315446	-0.174333	0.0355729	9.54861E-07	13	43122849
Malignant neoplasm of brain	rs7329344	A	T	0.33974	0.156655	0.0338662	3.73328E-06	13	114184250
Malignant neoplasm of brain	rs78378222	G	T	0.0166063	0.589882	0.106166	2.75689E-08	17	7668434
Brain glioblastoma	rs10069690	T	C	0.295524	0.465344	0.0690484	1.59038E-11	5	1279675
Brain glioblastoma	rs151057105	T	C	0.110556	0.531274	0.0938627	1.51269E-08	7	54877227
Brain glioblastoma	rs58495994	T	A	0.0668399	0.551781	0.117964	2.90315E-06	8	104165979
Brain glioblastoma	rs3217992	T	C	0.38625	-0.397531	0.0715297	2.73577E-08	9	22003224
Brain glioblastoma	rs113726896	T	C	0.0271713	0.760583	0.163967	3.50736E-06	22	38263316
Benign neoplasm: Brain, supratentorial	rs12127787	C	T	0.239155	0.295186	0.0646238	4.92969E-06	1	88993078
Benign neoplasm: Brain, supratentorial	rs12489876	A	G	0.00577852	1.21464	0.259618	2.88895E-06	3	20548759
Benign neoplasm: Brain, supratentorial	rs114377769	A	G	0.0283651	-1.12324	0.242905	3.76045E-06	3	31684560
Benign neoplasm: Brain, supratentorial	rs2114070	T	A	0.293196	0.290672	0.0611921	2.03264E-06	11	108958631
Benign neoplasm: Brain, supratentorial	rs117009158	A	G	0.0226995	0.716979	0.155067	3.77016E-06	16	10628419
Benign neoplasm: Brain, supratentorial	rs74432758	A	C	0.0585257	0.527395	0.109338	1.41043E-06	18	5987943
Benign neoplasm: Brain, supratentorial	rs34855348	A	G	0.216338	-0.346395	0.074809	3.64972E-06	19	41028010
Benign neoplasm: Brain, supratentorial	rs56287684	T	A	0.00100773	2.24278	0.420471	9.6086E-08	21	42706518
Benign neoplasm: Brain, infratentorial	rs143181153	A	T	0.0247413	1.03431	0.217831	2.05215E-06	4	35173902
Benign neoplasm: Brain, infratentorial	rs13103825	C	A	0.138297	0.554667	0.118851	3.05752E-06	4	58564275
Benign neoplasm: Brain, infratentorial	rs150063946	A	G	0.00658719	1.67922	0.346196	1.23154E-06	4	172156126
Benign neoplasm: Brain, infratentorial	rs142987224	G	A	0.0492949	0.808556	0.17086	2.22019E-06	5	59011095
Benign neoplasm: Brain, infratentorial	rs115080749	A	G	0.0144182	1.22055	0.264084	3.80391E-06	14	50407722

SNP, identifier for the specific Single Nucleotide Polymorphism; beta, effect size of the SNP on the outcome

Supplementary Table 6. Mendelian randomization estimates for the causal effect of genetic liability to brain tumor on T. gondii antibody

exposure	outcome	method	nsnp	b	se	pval	lo_ci	up_ci	or	or_lci95	or_uci95
Brain meningioma	P22	MR Egger	18	-0.054331907	0.12786546	0.676558116	-0.304948208	0.1962844	0.9471177	0.7371616	1.2168729
Brain meningioma	P22	Weighted median	18	-0.021759059	0.075223991	0.772384789	-0.169198081	0.12568	0.978476	0.8443416	1.1339192
Brain meningioma	P22	Inverse variance weighted	18	0.018424089	0.055398189	0.739454623	-0.090156361	0.1270045	1.0185949	0.9137883	1.1354222
Brain meningioma	P22	Simple mode	18	-0.029710773	0.138281643	0.832435176	-0.300742794	0.2413212	0.9707263	0.7402682	1.2729299
Brain meningioma	P22	Weighted mode	18	-0.007899379	0.129860435	0.952204208	-0.262425831	0.2466271	0.9921317	0.7691834	1.2797018
Brain meningioma	SAG1	MR Egger	18	0.017135664	0.073528643	0.818679727	-0.126980477	0.1612518	1.0172833	0.8807509	1.1749808
Brain meningioma	SAG1	Weighted median	18	0.006036486	0.044099328	0.891122609	-0.080398197	0.0924712	1.0060547	0.9227488	1.0968815
Brain meningioma	SAG1	Inverse variance weighted	18	0.009256874	0.03244854	0.775430702	-0.054342264	0.072856	1.0092999	0.9471079	1.0755757
Brain meningioma	SAG1	Simple mode	18	0.052346669	0.076836221	0.504872359	-0.098252323	0.2029457	1.053741	0.9064202	1.2250059
Brain meningioma	SAG1	Weighted mode	18	0.024282247	0.069832294	0.732316889	-0.11258905	0.1611535	1.0245795	0.8935178	1.1748653
Malignant neoplasm of brain	P22	MR Egger	10	0.219046697	0.193689115	0.290850505	-0.160583967	0.5986774	1.2448894	0.8516463	1.8197104
Malignant neoplasm of brain	P22	Weighted median	10	0.146017144	0.093108984	0.116825355	-0.036476464	0.3285108	1.157216	0.9641808	1.3888982
Malignant neoplasm of brain	P22	Inverse variance weighted	10	0.21033381	0.073463255	0.004194972	0.06634583	0.3543218	1.2340899	1.0685962	1.4252137
Malignant neoplasm of brain	P22	Simple mode	10	0.124217432	0.137894694	0.391155039	-0.146056169	0.394491	1.132262	0.8641092	1.4836289
Malignant neoplasm of brain	P22	Weighted mode	10	0.126030395	0.125878553	0.342883946	-0.120691568	0.3727524	1.1343166	0.8863073	1.4517248
Malignant neoplasm of brain	SAG1	MR Egger	10	-0.01376112	0.121264985	0.912446335	-0.251440491	0.2239183	0.9863331	0.7776797	1.2509687
Malignant neoplasm of brain	SAG1	Weighted median	10	0.107659376	0.057431441	0.060851615	-0.004906248	0.220225	1.1136683	0.9951058	1.2463571
Malignant neoplasm of brain	SAG1	Inverse variance weighted	10	0.090458324	0.045739527	0.047964381	0.000808852	0.1801078	1.0946759	1.0008092	1.1973464
Malignant neoplasm of brain	SAG1	Simple mode	10	0.015201635	0.094200759	0.875362672	-0.169431852	0.1998351	1.0153178	0.8441443	1.2212014
Malignant neoplasm of brain	SAG1	Weighted mode	10	0.127514635	0.084541606	0.165750017	-0.038186913	0.2932162	1.1360015	0.962533	1.3407326
Brain glioblastoma	P22	MR Egger	5	-0.261896182	0.281729645	0.421147937	-0.814086285	0.2902939	0.7695909	0.443044	1.3368204
Brain glioblastoma	P22	Weighted median	5	0.021405852	0.064116993	0.73848812	-0.104263454	0.1470752	1.0216366	0.9009879	1.158441
Brain glioblastoma	P22	Inverse variance weighted	5	0.003655038	0.053351693	0.945380954	-0.100914281	0.1082244	1.0036617	0.9040105	1.1142977
Brain glioblastoma	P22	Simple mode	5	0.041910678	0.085127318	0.648284991	-0.124938865	0.2087602	1.0428013	0.8825509	1.2321495
Brain glioblastoma	P22	Weighted mode	5	0.036650235	0.077388039	0.66048237	-0.115030322	0.1883308	1.0373301	0.8913391	1.2072328
Brain glioblastoma	SAG1	MR Egger	5	0.141139883	0.161444241	0.446338079	-0.175290829	0.4575706	1.1515857	0.8392129	1.5802303
Brain glioblastoma	SAG1	Weighted median	5	0.033196833	0.039962521	0.406143593	-0.045129708	0.1115234	1.033754	0.9558735	1.1179799
Brain glioblastoma	SAG1	Inverse variance weighted	5	0.032089087	0.030891438	0.298911886	-0.02845813	0.0926363	1.0326095	0.971943	1.0970627
Brain glioblastoma	SAG1	Simple mode	5	0.055179119	0.056401412	0.383306104	-0.055367648	0.1657259	1.0567299	0.9461372	1.1802495
Brain glioblastoma	SAG1	Weighted mode	5	0.06415856	0.054895639	0.307410582	-0.043436891	0.171754	1.0662615	0.957493	1.1873857
Benign neoplasm: Brain, supratentorial	P22	MR Egger	7	0.074780513	0.170354295	0.679003782	-0.259113904	0.4086749	1.0776476	0.7717351	1.5048225
Benign neoplasm: Brain, supratentorial	P22	Weighted median	7	0.031548881	0.073154032	0.666274627	-0.111833021	0.1749308	1.0320518	0.8941936	1.1911638
Benign neoplasm: Brain, supratentorial	P22	Inverse variance weighted	7	0.045290448	0.07033706	0.519636457	-0.09257019	0.1831511	1.0463317	0.9115852	1.2009958
Benign neoplasm: Brain, supratentorial	P22	Simple mode	7	-0.004384867	0.101445917	0.966925768	-0.203218864	0.1944491	0.9956247	0.8160996	1.2146417
Benign neoplasm: Brain, supratentorial	P22	Weighted mode	7	-0.007463034	0.094596931	0.939683123	-0.192873018	0.177947	0.9925647	0.8245867	1.1947619
Benign neoplasm: Brain, supratentorial	SAG1	MR Egger	7	-0.036683801	0.112007012	0.756547863	-0.256217545	0.1828499	0.9639809	0.7739736	1.2006342
Benign neoplasm: Brain, supratentorial	SAG1	Weighted median	7	0.063906108	0.04424891	0.148671078	-0.022821755	0.150634	1.0659923	0.9774367	1.162571
Benign neoplasm: Brain, supratentorial	SAG1	Inverse variance weighted	7	0.01076357	0.04614275	0.815554265	-0.079676221	0.1012034	1.0108217	0.9234153	1.1065016
Benign neoplasm: Brain, supratentorial	SAG1	Simple mode	7	0.093016378	0.064767861	0.200979033	-0.033928629	0.2199614	1.0974797	0.9666405	1.2460286
Benign neoplasm: Brain, supratentorial	SAG1	Weighted mode	7	0.089573669	0.061917008	0.198133978	-0.031783666	0.210931	1.0937079	0.9687161	1.2348272
Benign neoplasm: Brain, infratentorial	P22	MR Egger	5	-0.125676059	0.270754868	0.67413517	-0.656355601	0.4050035	0.8819005	0.5187384	1.4993077
Benign neoplasm: Brain, infratentorial	P22	Weighted median	5	0.020932644	0.080423938	0.794648741	-0.136698274	0.1785636	1.0211533	0.8722334	1.1954989
Benign neoplasm: Brain, infratentorial	P22	Inverse variance weighted	5	0.047620574	0.099887378	0.633545381	-0.148158686	0.2433998	1.0487726	0.8622943	1.2755785
Benign neoplasm: Brain, infratentorial	P22	Simple mode	5	0.004632219	0.097081074	0.964230747	-0.185646687	0.1949111	1.004643	0.830567	1.215203
Benign neoplasm: Brain, infratentorial	P22	Weighted mode	5	-0.00254893	0.090207844	0.978811381	-0.179356305	0.1742584	0.9974543	0.835808	1.1903632
Benign neoplasm: Brain, infratentorial	SAG1	MR Egger	5	0.052350941	0.10180667	0.642580784	-0.147190133	0.251892	1.0537455	0.8631299	1.2864571
Benign neoplasm: Brain, infratentorial	SAG1	Weighted median	5	-0.033522642	0.043738512	0.44341866	-0.119250124	0.0522048	0.967033	0.8875858	1.0535915
Benign neoplasm: Brain, infratentorial	SAG1	Inverse variance weighted	5	-0.049864103	0.040502668	0.218273754	-0.129249332	0.0295211	0.9513587	0.8787548	1.0299612
Benign neoplasm: Brain, infratentorial	SAG1	Simple mode	5	-0.007180765	0.070928623	0.924232214	-0.146200866	0.1318393	0.992845	0.8639841	1.140925
Benign neoplasm: Brain, infratentorial	SAG1	Weighted mode	5	-0.000949801	0.058310164	0.987784096	-0.115237723	0.1133381	0.9990506	0.8911543	1.1200106

MR, mendelian randomization; *T. gondii, Toxoplasma gondii*; nsnp, number of single-nucleotide polymorphisms (SNPs) used in the analysis; pval, p-value; or, odds ratio representing the magnitude of the causal effect; or_lci95, lower bound of the 95% confidence interval for the odds ratio; or_uci95, upper bound of the 95% confidence interval for the odds ratio

Supplementary Table 7. Pleiotropy assessment of brain tumor on T. gondii antibody seropositivity using the MR-Egger intercept test.

exposure	outcome	egger_intercept	se	pval
Brain meningioma	P22	0.020677386	0.032751859	0.536737591
Brain meningioma	SAG1	-0.002260266	0.018928751	0.906437676
Malignant neoplasm of brain	P22	-0.002178117	0.044802011	0.962416752
Malignant neoplasm of brain	SAG1	0.026111983	0.02810256	0.379984598
Brain glioblastoma	P22	0.129616137	0.135024625	0.407898927
Brain glioblastoma	SAG1	-0.05344392	0.077659126	0.540774491
Benign neoplasm: Brain, supratentorial	P22	-0.01386834	0.071518612	0.853876425
Benign neoplasm: Brain, supratentorial	SAG1	0.021946677	0.046484625	0.656722447
Benign neoplasm: Brain, infratentorial	P22	0.160585661	0.230469912	0.536081036
Benign neoplasm: Brain, infratentorial	SAG1	-0.094309058	0.086540843	0.355524675

T. gondii, Toxoplasma gondii; se, Standard error; Pval, p-value

Supplementary Table 8. Heterogeneity assessment of brain tumor on T. gondii antibody seropositivity using Cochran's Q test.

exposure	outcome	Q	Q_df	Q_pval
Brain meningioma	P22	13.65767284	17	0.691168364
Brain meningioma	SAG1	10.90130113	17	0.861648555
Malignant neoplasm of brain	P22	3.367121665	9	0.947947162
Malignant neoplasm of brain	SAG1	10.35678424	9	0.322380956
Brain glioblastoma	P22	2.218776562	4	0.695593188
Brain glioblastoma	SAG1	2.511721097	4	0.642538186
Benign neoplasm: Brain, supratentorial	P22	9.982967186	6	0.125371143
Benign neoplasm: Brain, supratentorial	SAG1	12.30834642	6	0.055433063
Benign neoplasm: Brain, infratentorial	P22	9.797399541	4	0.043982308
Benign neoplasm: Brain, infratentorial	SAG1	5.701303917	4	0.222593179

T. gondii, Toxoplasma gondii
